# Pheromone-Mediated Social Organization and Pest Management of the Red Imported Fire Ant, *Solenopsis invicta*: A Review

**DOI:** 10.3390/insects17020150

**Published:** 2026-01-28

**Authors:** Mengbo Guo, Nazakat Osman, Shunhai Yu, Junyan Liu, Yiping Wang, Jianyu Deng

**Affiliations:** 1 Zhejiang Key Laboratory of Biology and Ecological Regulation of Crop Pathogens and Insects, College of Advanced Agricultural Sciences, Zhejiang Agricultural and Forestry University, Hangzhou 311300, China; guomengbo@zafu.edu.cn (M.G.);; 2 Qianjiangyuan National Park Administration, Kaihua 324300, China; 3 College of Forest and Biotechnology, Zhejiang Agricultural and Forestry University, Hangzhou 311300, China

**Keywords:** *Solenopsis invicta*, pheromone communication, chemical ecology, social organization, pest management

## Abstract

Pheromone-mediated chemical communication plays a central role in shaping the social organization and ecological success of *S. invicta*, a globally invasive eusocial insect characterized by a highly developed semiochemical signaling system. This review summarizes recent advances in the chemical ecology of *S. invicta*, with emphasis on the putative ecological roles of major pheromone classes, current understanding of the molecular and neurobiological basis of pheromone perception and signal processing, and the associations between chemical cues and colony-level social behavior dynamics. Furthermore, we evaluate progress in pheromone-based management approaches, including pheromone-enhanced baits and trail disruption techniques, highlighting both their potential to improve the specificity and efficacy of fire ant management and the current practical limitations for large-scale field applications. Finally, current significant knowledge gaps and challenges are discussed, particularly the partial characterization of pheromone identity, the ambiguous and biological significance of chemical cues, and challenges in applying laboratory research in pest management under field conditions. By linking chemical ecology, neurobiology, and invasion biology to pest management, this review outlines priority directions for future research and provides a theoretical foundation for developing more sustainable, targeted pest control approaches for fire ant management.

## Simple Summary

The red imported fire ant, *Solenopsis invicta*, is one of the most aggressive invasive insects worldwide, causing economic and ecological damage. Their invasion success relies heavily on an efficient pheromone signaling system that regulates collective behaviors, including foraging, defense, nursing, and reproduction. This review summarizes current knowledge on how different pheromones regulate social organization and collective behaviors and discusses their potential applications in pest management, including pheromone-enhanced baits and behavioral disruption strategies. Current research gaps, challenges, and future directions are discussed to inform the development of more targeted, efficient, and sustainable pest management strategies.

## 1. Introduction

**Figure 1:**
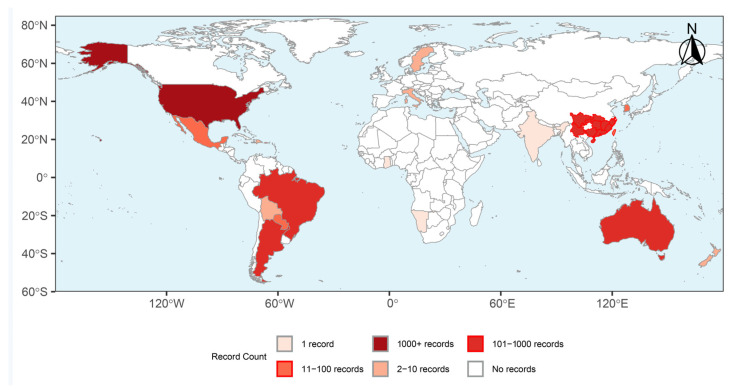
Global Distribution Map of *S. invicta*. The map was generated using occurrence data obtained from the Global Biodiversity Information Facility (GBIF) based on 14,401 “human observation” georeferenced records from 18 countries and territories. Color shading indicates the number of occurrence records per country or territory. “Record Count” reflects reporting frequency rather than actual population density or invasion intensity. GBIF Occurrence Download. Available online: https://doi.org/10.15468/dl.c44689 (accessed on 26 September 2025).

The red imported fire ant, *Solenopsis invicta* Buren (Hymenoptera: Formicidae), is a globally invasive species that has caused significant ecological and economic damage [1,2,3]. Since its introduction into the southern United States, *S. invicta* has been recorded in 18 countries and territories across five continents based on human-observation occurrence records (Figure 1) [2]. As a eusocial insect, *S. invicta* exhibits highly organized social behavior, with a complex social hierarchy and differentiated caste. A sophisticated pheromone communication system is widely considered to play an important role in mediating a wide range of social behaviors in *S. invicta*, including foraging, recruitment, defense, nest maintenance, brood nursing, reproductive regulation, and nestmate recognition [4]. Understanding the mechanisms of pheromone-mediated behavior regulation is crucial for developing more targeted management strategies for this invasive pest [5,6]. However, the practical large-scale application remains challenging due to limited understanding of pheromone properties and their ecological roles in complex social contexts, as well as practical and technical issues related to chemical synthesis, formulation, and field performance. This review summarizes current knowledge on the chemical ecology of pheromone communication in *S. invicta*, focusing on chemical identity, molecular and neural mechanisms of signal perception, intra- and interspecific behavior regulation, and recent advances and limitations in pheromone-based management strategies.

## 2. Social Organization and Pheromone Communication of *S*. *invicta*

### 2.1. Social Hierarchy Structure and Colony Forms

The fire ant colonies display notable characteristics of a “superorganism” with mature nests comprising thousands to hundreds of individuals that exhibit significant physiological and behavioral differentiation despite sharing a similar genetic background [7,8]. Individuals can achieve effective collaboration and behavioral integration through chemical communication and behavioral regulation, including foraging trail marking, alarm signaling, brood care, and nest maintenance [9,10,11]. A colony of *S. invicta* consists of a queen or multiple queens, workers, and males. The queen, the largest individual in the colony, is responsible exclusively for oviposition, while males, which appear seasonally during nuptial flights, mate with newly emerged queens and die shortly after copulation [12,13]. Worker ants, which constitute the most numerous caste in a colony, perform a wide range of tasks, including foraging, brood care, nest construction, and colony defense [14,15].

Two distinct social forms have been identified in *S. invicta*: the monogyne with single-queen and polygyne with multiple-queen colonies [15,16]. In a monogyne colony, reproduction is monopolized by a single queen [17]. Individuals in the nest are highly exclusive and exhibit strong aggression toward non-nestmates [18,19]. The colony expands relatively slowly and exhibits greater structural stability [20]. In contrast, polygyne colonies accommodate multiple queens, and members show less exclusivity and aggression [17,18].

### 2.2. Functional Classification of Pheromone Systems in S. invicta

*S. invicta* employs a sophisticated chemical communication system that contributes to social organization and collective activities [21]. Pheromone and related chemical cues in *S. invicta* can be grouped into several functional categories (Table 1), ranging from well-characterized trail and alarm pheromones to candidate queen-associated cues and nestmate-related semiochemical signals.

Trail and alarm pheromones have been chemically characterized with defined active components, and consistently elicit trail-following or recruitment behaviors in laboratory and field assays, supporting their roles in collective foraging and colony defense. Trail pheromones, primarily secreted from the Dufour’s gland, guide workers to food sources and maintain foraging trails [22]. Alarm pheromones, with 2-ethyl-3,6-dimethylpyrazine as the major component, trigger defensive responses and recruit nestmates [23]. Queen-associated signals involve a diverse set of chemical cues from different sources, including glandular secretions and cuticular hydrocarbons (CHCs) [24,25,26]. Behavioral evidence suggests that some of these cues can influence worker attraction, retinue behavior, and reproductive regulation at the colony level. However, the specific signaling roles and underlying chemical identities remain unresolved [24,25,26]. Brood- and nestmate-related cues in *S. invicta* are majorly contact-based chemical signals that are closely associated with task allocation and nestmate recognition. Current knowledge of the functional evidence and underlying mechanisms supporting these pheromone systems is discussed in detail in subsequent sections.

**Table 1:** Functional Classification of *S. invicta* Pheromones.

Types	Functional Evidence	Components	Source	References
Trail pheromone	Foraging recruitment and trail maintenance	Z,E-α-farnesene E,E-α-farnesene Z,E-α-homofarnesene E,E-α-homofarnesene	Dufour’s gland	[27,28]
Alarm pheromone	Alarm signaling and defensive recruitment	2-Ethyl-3,6-dimethylpyrazine (principal component)	Mandibular glands	[23]
Queen-associated cues	Worker attraction, retinue behavior	(E)-6-(1-pentenyl)-2H-pyran-2-one Invictolide Dihydroactinidiolide	Venom sac	[24,29]
	Promote egg-tending behavior	Unidentified compounds	Venom sac	[25]
	reproductive suppression and sexual larvae execution	Unidentified compounds	Venom sac/postpharyngeal glands	[26]
Nestmate recognition pheromones	Nestmate discrimination	Colony-specific blends of CHCs	Worker cuticle	[30,31]

## 3. Molecular and Neural Basis of Pheromone Communication in *S. invicta*

**Figure 2:**
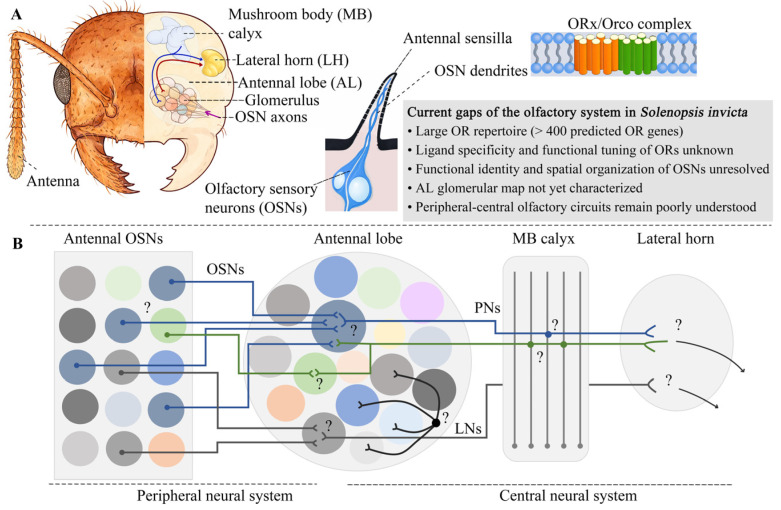
Schematic Overview of the Insect Olfactory System and Current Knowledge Gaps in *S. invicta*. (**A**) Simplified schematic of the olfactory system of *S. invicta*. Odorant molecules are detected by the OR/Orco complex expressed on the dendritic membranes of OSNs housed within antennal sensilla. Axons of OSNs project to the AL, where they terminate in discrete glomeruli. Processed olfactory information is subsequently transmitted to higher-order brain centers, including the MB calyx and the LH. The boxed text summarizes major knowledge gaps in the olfactory system of *S. invicta*. (**B**) Conceptual map of the insect olfactory neural system. Arrows represent putative directions of signal transmission. Different colors are used for visualization to denote distinct antennal OSN populations, glomeruli within the AL, and neuron pathways. Question marks (“?”) indicate unknown or uncharacterized neuronal identities, spatial organization, and synaptic connections in peripheral and central olfactory systems, and the behavioral relevance of these neural pathways in *S. invicta*. The schematic is adapted from Zhao et al. (2020) [37].

In insects, pheromone components are mainly detected by a sensitive olfactory system that employs several families of olfactory proteins and sophisticated peripheral and central neural circuits [32]. A typical process of olfactory perception includes the following steps: odorant molecules first enter the sensilla on the antenna and are bound to soluble carrier proteins such as odorant-binding proteins (OBPs) in the sensillar lymph and transported to olfactory sensory neurons (OSNs) [32]. Then, the ligands bind to specific odorant receptors (ORs) expressed on the dendritic membranes of OSNs, where the chemical information carried by the odorant molecule is transduced into a nervous impulse and then conveyed by OSN axons to the antennal lobe (AL), the primary olfactory processing center in the insect brain (Figure 2A) [33]. Within the AL, axon terminals of different OSN types converge onto discrete glomeruli, each of which typically receives input from OSNs expressing the same type of OR, forming a one-to-one mapping and constituting a parallel channel that represents an identity and intensity map of peripheral odor [34]. The glomeruli synapse with local neurons (LNs) and projection neurons (PNs), thereby transmitting processed information from AL to higher brain centers such as the lateral horn (LH) and the mushroom body (MB) calyx for further integration [35,36]. The peripheral and central neural circuits constitute the framework for pheromone perception, enabling olfactory signals to be detected, encoded, and integrated across multiple levels of the nervous system.

### 3.1. Olfactory Proteins Underlying Peripheral Pheromone Detection

Specific OR, which is typically co-expressed with the conserved co-receptor Orco, is considered one of the molecular switches of the peripheral olfactory system [32]. The function of OSNs to encode odor recognition is determined by the specific functional characteristics of the OR/Orco heteromeric complex expressed on the dendritic membranes [32]. Studies have shown that ORs play essential roles in both neural development and the regulation of social behavior in ants. In the clonal raider ant *Ooceraea biroi*, researchers demonstrated that loss of the *Orco* gene significantly reduced the olfactory sensilla number on the antennae, and the majority of glomeruli disappeared or disorganized in the AL [38]. Furthermore, social behaviors such as nestmate recognition, brood care, and foraging are profoundly impaired in *Orco* mutants, leading to the collapse of colony organization due to the inability to maintain coordinated social functions [38].

The size and functional specialization of the OR repertoire in one species fundamentally shape the ability to perceive chemical cues. Genomic studies have revealed that *S. invicta* possesses one of the most extensive OR gene repertoires reported in insects (Table 2, representative species from major insect orders). Genome annotation in *S. invicta* annotated a large OR repertoire with 297–356 intact genes, revealing a striking expansion of OR repertoire [7,39]. Notably, a substantial proportion of these expanded genes belongs to the 9-exon OR subfamily, which is considered to be derived from an ancestral lineage in Hymenoptera and is predominantly expanded in ant species [39]. In *S. invicta*, ~25% of ORs belong to the 9-exon OR clade, with ~85% of these genes organized into gene clusters on specific chromosomes, suggesting that the expansion of these genes may have largely derived by local gene duplication [39].

**Table 2:** Odorant Receptor (OR) Gene Repertoires Across Insect Orders Based on Genome Annotation.

Order	Species	Gene Number	Reference
Hymenoptera	*Solenopsis invicta*	356 intact ORs	[39]
	*Ooceraea biroi*	298 intact ORs	[39]
	*Monomorium pharaonis*	306 intact ORs	[39]
	*Harpegnathos saltator*	347 intact ORs	[40]
	*Acromyrmex echinatior*	385 ORs	[41]
	*Apis mellifera*	170 ORs	[42]
	*Telenomus elegans*	260 intact ORs	[43]
	*Telenomus remus*	46 intact ORs	[43]
Coleoptera	*Rhynchophorus ferrugineus*	80 ORs	[44]
	*Anoplophora glabripennis*	98 ORs	[45]
Diptera	*Drosophila melanogaster*	62 ORs	[46]
	*Anopheles gambiae*	79 ORs	[47]
Orthoptera	*Locusta migratoria*	95 ORs	[48]
	*Gryllotalpa orientalis*	30 ORs	[49]
Hemiptera	*Acyrthosiphon pisum*	79 ORs	[50]
	*Riptortus pedestris*	237 ORs	[51]
Blattodea	*Blattella germanica*	105 intact ORs	[52]
	*Zootermopsis nevadensis*	80 intact ORs	[53]
Lepidoptera	*Manduca sexta*	73 ORs	[54]
	*Spodoptera frugiperda*	69 ORs	[55]
Phthiraptera	*Pediculus humanus* humanus	10 ORs	[56]

Evolutionary analyses revealed heterogeneous selection pressures among 9-exon OR clusters, with only a subset of gene clusters showing signatures of accelerated evolution under positive selection, whereas others remain relatively conserved [39]. Expression analyses showed that multiple 9-exon ORs are highly and preferentially expressed in worker antennae, consistent with a role in detecting social chemical cues [39]. However, whether these rapidly evolving ORs contribute to species-specific behaviors in *S. invicta* remains unknown. Functional characterization in other ant species, such as the ponerine ant *Harpegnathos saltator*, has revealed a strong bias of 9-exon ORs toward CHCs, despite CHC detection not being restricted to this subfamily [57,58].

In addition to ORs, other classes of olfactory proteins have been reported to contribute to peripheral pheromone detection in *S. invicta*. An antenna-specific OBP, SiOBP5, was reported to be required for sensing bait-associated odorants, highlighting its role in foraging-related cues [59]. More recently, an antennal-expressed Niemann-Pick type C2 (NPC2) protein was shown to participate in the detection of alarm pheromones in *S. invicta* workers [60]. Despite these advances, our understanding of peripheral pheromone detection in *S. invicta* remains limited. To date, systematic functional characterization of olfactory proteins, particularly ORs, remains largely lacking, and no OR-ligand pair has been validated. This gap significantly limits our understanding of how specific receptors contribute to pheromone detection and colony-level social behaviors.

### 3.2. Neural Processing and Coding Strategies of Pheromone Signals

OSNs constitute the fundamental functional units for pheromone detection, whereas the antennal sensilla in which they are housed represent the basic structural units of the peripheral olfactory system. In *S. invicta*, the functional types of OSNs and their spatial organization have not yet been characterized, while morphological studies have revealed pronounced differences in morphology and distribution among sexes and castes, which may provide a structural basis for differential pheromone perceptions [61]. After being detected by OSNs, pheromone molecules are conveyed into the AL and then transmitted to higher-order brain centers (Figure 2). The organization and coding strategies of the olfactory nervous system play a central role in determining how pheromone cues are represented, filtered, integrated, and translated into coordinated individual and colony-level responses [32]. In *S. invicta*, research on the olfactory neural circuits remains limited (Figure 2B). To date, there are no direct documents on glomerular architecture and coding strategies of pheromone signals at different neural processing levels.

Studies in some other ant species indicate that the AL structure is more elaborate than that in other hymenopterans, with exceedingly large numbers of glomeruli, consistent with the extensive expansion of OR genes [62,63]. In *O. biroi*, neuroanatomical and functional imaging analyses revealed a modular organization and functional differentiation in AL, in which alarm pheromones are encoded by sparse, highly stereotyped activation of a small subset of glomeruli, including a core glomerulus that is reliably activated across individuals [33,63]. Such an organization is considered to be critical for encoding signals related to social behavior, as stereotyped coding enables faithful behavioral responses across individuals, thereby promoting rapid collective decisions at the colony level [64].

Beyond fixed neural circuits, pheromone processing in social insects can be modulated by neuroendocrine signals. A recent work in *O. biroi* demonstrated that the neural representation of alarm pheromones in the AL changes with age, with no detectable changes in peripheral sensory structures, suggesting that neuromodulators may contribute to age-dependent plasticity [65]. In *S. invicta*, direct evidence for neuromodulators modulating pheromone signaling remains limited and focuses primarily on correlations between behavior and physiology. Research on biogenic amines has shown that the presence or absence of a queen within the colony significantly alters octopamine levels in worker brains, which in turn influence social behaviors, including nestmate recognition and aggression [66]. Studies on sNPF revealed that its receptor is broadly expressed in regions associated with olfactory processing, and its expression patterns are caste-dependent in workers [67,68]. While these works above do not establish how such signals modulate pheromone perception and representation within olfactory neural circuits. To date, the neural architecture and coding strategies underlying pheromone processing in *S. invicta* have not been directly characterized. Future studies linking neuromodulatory signaling to neural dynamics within olfactory circuits will be critical for elucidating how pheromone communication is flexibly regulated by colony context and individual physiological state

## 4. Intraspecific Behavioral Regulation Mediated by Pheromone

**Figure 3:**
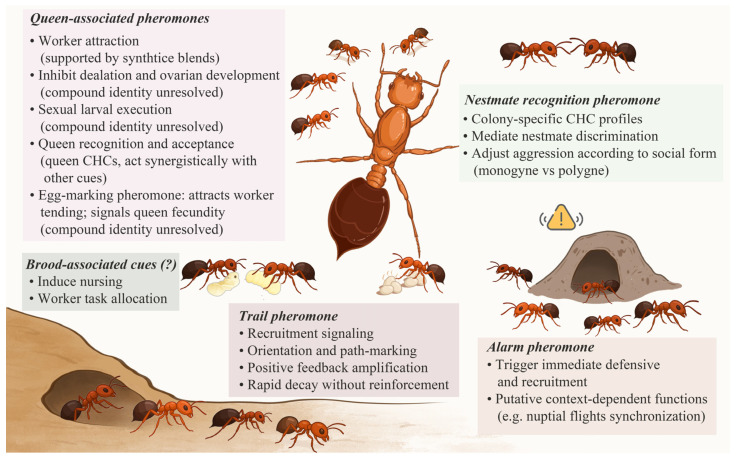
Pheromone-mediated Behavioral Regulation in *S. invicta*. The schematic summarizes the major categories of pheromones and pheromone-associated cues involved in intraspecific behavioral regulation in *S. invicta*. The schematic was created by the authors based on published literature and is intended as a conceptual summary of current knowledge presented in Section 4.1, Section 4.2, Section 4.3, Section 4.4. Question mark (“?”) indicates that the presence of this class of pheromones remains controversial. Background colors denote functional categories for visualization. The warning icon highlights the role of alarm pheromones in triggering rapid defensive and collective responses in *S. invicta*.

Within *S. invicta* colonies, virtually all social behaviors, including foraging, defense, brood care, reproduction, and maintenance of reproductive hierarchy, are regulated by pheromone communication [4,69]. By releasing specific chemical signals, fire ants can elicit immediate behavioral responses such as trail-following and alarm recruitment as well as induce long-term physiological changes, such as reproductive suppression and brood management [21,69]. Below, we describe the major pheromonal systems in *S. invicta* and summarize current documents for their roles in regulating intraspecific behavior, highlighting how they reinforce social structure and enable adaptive colony-level responses (Figure 3).

### 4.1. Trail and Alarm Pheromones: Collective Foraging and Defense

Trail and alarm pheromones are among the core social signals that coordinate rapid collective behaviors for resource acquisition and colony defense in *S. invicta*. Trail pheromones guide workers along defined paths from the nest to food resources or new nesting sites, with scout workers depositing a pheromone trail on the path to attract and recruit more workers for foraging activity [22,27]. Chemical analyses indicate that trail pheromones are produced primarily by the Dufour’s gland and consist of a blend of sesquiterpenes, including Z,E-α-farnesene, E,E-α-farnesene, Z,E-α-homofarnesene, and E,E-α-homofarnesene. Although early studies identified Z,Z,Z-allofarnesene as the major trail pheromone component, subsequent behavioral studies failed to verify the trail-following activity [27,70]. A more recent study demonstrated Z,E-α-farnesene, and E,E-α-farnesene are the primary active components eliciting trail-following responses in worker ants [22]. Trail pheromones are highly species-specific and effective, allowing fire ants to distinguish conspecific trails from those of other species, and only trace amounts can elicit rapid recruitment [71]. Beyond guiding foraging, trail pheromones dynamically regulate the recruitment processes through positive feedback: workers repeatedly deposit and reinforce the pheromone trail along the foraging path toward a high-quality food source, and the chemical cues fade naturally once the food source is depleted or the trail cues are no longer reinforced [28,72,73]. This recruitment strategy provides *S. invicta* with advantages over native competitors by enabling high foraging efficiency and flexibility.

Alarm pheromones majorly coordinate rapidly collective defensive responses when a colony is disturbed or threatened [74]. A pyrazine compound, 2-ethyl-3,6-dimethylpyrazine, produced by the mandibular glands was identified as the key component that induces heightened alertness in nearby worker ants, including rapid movement, mandible opening, and stinging behavior [23,74]. When at an appropriate higher concentration, the alarm pheromone can trigger mass recruitment, with workers swarming out of the nest to confront intruders collectively [75]. Beyond defense, alarm pheromones have also been suggested to act in other behaviors. For example, studies have revealed caste- and sex-specific differences in alarm pheromone production, with virgin queens and winged male ants producing significantly higher quantities than workers, suggesting a potential role in coordinating group behavior prior to nuptial flights [74]. However, direct behavioral evidence has not yet been reported. Together, trail and alarm pheromones enable rapid, flexible coordination by amplifying individual chemical signals into colony-level responses, thereby supporting efficient foraging and collective defense.

### 4.2. Queen-Associated Pheromones and Reproductive Regulation

Queens of *S. invicta* produce complex pheromone blends that regulate colony organization by both releaser and primer effects. Releaser pheromones typically immediately attract workers for tending and care. In contrast, primer pheromones induce long-term physiological effects such as suppressing ovarian development in workers and regulating the development of new sexual individuals [76,77]. Early studies demonstrated that inanimate objects with the volatiles and pentane extracts of the mated queen’s venom sac are highly attractive to workers and promote the deposition of brood [78,79]. Subsequent works isolated at least three candidate active components: (E)-6-(1-pentenyl)-2H-pyran-2-one, invictolide (tetrahydro-3,5-dimethyl-6-(1-methylbutyl)-2H-pyran-2-one), and dihydroactinidiolide, and the blend of which acts as a strong releaser effect on workers by inducing aggregation and retinue behavior around treated surrogate queens [24,29]. However, there is no further evidence demonstrating that these compounds function as specific signals for queen identity or caste recognition.

Beyond these immediate releaser effects, queens also produce pheromones that regulate the reproduction of the colony level through primer effects. An early study reported the presence of an egg-marking pheromone in the queen’s venom sac, which is applied onto the eggs during oviposition and was shown to attract workers, promote egg-tending behavior, and facilitate aggregation around the queen [25]. By contacting and handling the eggs, workers can perceive the flux of the pheromone cue, which is directly correlated with the queen’s fecundity [25]. In addition, the extract from queens’ venom sac has been documented to regulate the reproduction on colony-level by inducing workers to execute sexual larvae, which survive in queenless colonies, but are consistently killed in the presence of a dealated queen, and extracts of the venom gland were sufficient to trigger the execution behavior, thereby eliminating potential rival queens [26]. Although the active chemical molecules remain unidentified, this behavioral evidence indicates the functions of queen-derived glandular secretions in suppressing reproductive competition among both workers and developing sexual brood, thereby reinforcing the queen’s reproductive monopoly, stabilizing the division of labor, and reinforcing the social hierarchy within the colony.

### 4.3. Task Allocation of Workers and Colony Maintenance

In eusocial insects, efficient task allocation and cooperation among workers, including brood care, nest maintenance, and foraging, are essential for colony stability. In particular, chemical cues associated with developing brood play a central role in mediating worker responses to brood presence and stage-specific demands. In the honey bee *Apis mellifera*, brood pheromones have been characterized as brood-derived chemical cues that regulate worker division of labor and contribute to the suppression of worker reproduction, thereby allowing workers to adjust brood-care behavior in accordance with the stage-specific nutritional and physiological demands of developing larvae [80,81]. In ants, however, the existence of brood pheromone remains controversial.

An early study in *S. invicta* proposed the existence of a brood pheromone, which was isolated from the sexual brood and identified triolein as the major active component that elicited brood-tending behaviors in workers [82]. However, this viewpoint was subsequently challenged by noting that the bioassays failed to exclude food-related or nestmate recognition responses and argued that triolein was mistakenly identified as using inappropriate chemical techniques [83]. Subsequent studies have failed to provide clear evidence for a dedicated chemical cue produced by immature stages that specifically regulates worker behavior, instead supporting a multimodal model in which worker responses to brood depend on contact chemical cues (mainly CHCs), morphological traits, behavioral interactions, and experience [84,85].

### 4.4. Social Organization and Colony Boundaries Mediated by Pheromonal Cues

In ants, nestmate recognition is crucial for maintaining colony stability by enabling workers to discriminate nestmates from non-nestmates and thereby regulate acceptance, exclusion, and aggression at colony boundaries [86]. In most ant species, nestmate discrimination relies on chemical cues, primarily CHCs present on the cuticle of both brood and adult individuals. *S. invicta* has served as a key model system for studying the mechanisms underlying nestmate recognition. Studies have shown that each colony possesses a specific CHC profile, which provides the chemical basis for discrimination [87,88]. Workers perceive the CHC signature of encountered individuals, compare it with an internal colony-specific recognition template stored in the brain, and, when the degree of chemical similarity falls below a threshold, initiate exclusion or aggressive responses [89]. Notably, the CHC profiles of *S. invicta* colonies are dynamic rather than stereotyped, indicating that recognition templates are plastic and can be continuously updated through interactions among nestmates in response to changes in environmental conditions or colony structure [90]. This dynamic adjustment helps maintain accurate nestmate recognition and prevent misidentification, thereby promoting stability of social structure.

Nestmate recognition in monogyne and polygyne colonies of *S. invicta* has been examined to understand how colony boundaries are maintained under different social organizations. Monogyne colonies are typically characterized by strong territoriality and high levels of aggression toward non-nestmates, resulting in clearly defined colony boundaries [91]. In contrast, polygyne colonies have traditionally been considered to have less clearly defined boundaries and exhibit more diffuse colony boundaries, with workers from neighboring nests exhibiting more similar CHC signatures [30]. However, an experimental study indicates that although reduced aggression, polygyne colonies also maintain colony boundaries with workers still strongly discriminating against non-nestmate brood, resulting in reduced survival of non-nestmate larvae [31]. These findings suggest that the social organization and colony boundaries of *S. invicta* are likely maintained through the combined effects of genetic structure and nestmate recognition.

Genetic studies indicate that the social polymorphism in *S. invicta* is controlled by a large supergene located on a “social chromosome,” which contains a nonrecombining region comprising ~504 genes with two distinct haplotypes, SB and Sb. Monogyne queens are homozygous (SB/SB), whereas polygyne queens are heterozygous (SB/Sb) [92]. A more recent study has shown that the social supergene (Sb) of *S. invicta* not only determines social form but also modulates the CHC composition of the queen, which acts synergistically with fertility-related cues to regulate worker recognition and acceptance [91]. Nevertheless, worker responses toward queens are not fixed but are flexible at the colony level. Zeng et al. (2025) demonstrated that introducing SB/Sb workers into monogyne colonies can induce a conversion from monogyne to polygyne colony social form through a minority influence effect [30]. Specifically, when 10–20% SB/Sb workers are present, constitutive cues associated with the Sb supergene on their cuticle disseminate throughout the colony, thereby reducing the aggression of host workers toward the SB/Sb queen. Upon detecting a same-genotype queen, SB/Sb workers release inducible pheromones that trigger a behavioral cascade in host SB/SB workers, leading to acceptance of the SB/Sb queen [30]. These findings indicate that the social form of *S. invicta* colonies is shaped not only by underlying genotypes but also by flexible pheromonal communication among individuals, enabling plastic modulation of colony behavior and social organization.

## 5. Interspecific Chemical Interactions and Ecological Adaptation

Beyond regulating colony-level behaviors, semiochemical communication also plays a central role in mediating interspecific interactions between *S. invicta* with other organisms, including mutualistic hemipterans, specialized natural enemies, and pathogenic fungi. The related chemical cues function as mediators that shape cooperation, competition, enemy avoidance, and disease resistance, thereby contributing to the ecological adaptation of *S. invicta*.

### 5.1. Ant-Hemipteran Mutualism Mediated by Semiochemicals

In addition to competitive interactions, *S. invicta* evolves mutualistic relationships with certain phytophagous insects such as aphids, scale insects, and mealybugs [28,93]. For example, *S. invicta* workers protect aphids from natural enemies, and in return, they receive nutritionally rich honeydew as a reward, with chemical cues playing a central role in mediating these interactions [21,94]. Studies have shown that *S. invicta* can detect the alarm pheromone of aphids, E-β-farnesene, which acts as a signal for help, triggering increased patrolling and defensive activity of worker ants on the host plant [21]. In parallel, honeydew also contains volatile chemicals that attract foraging workers, who deposit trail pheromones to reinforce the food cue and recruit additional nestmates to the site after locating a honeydew source [28]. In addition to providing protection, *S. invicta* can manipulate the population dynamics of aphids to maintain a stable honeydew supply by using trail pheromones to suppress the production and dispersal of winged aphids [28]. This chemical regulation ensures a continuous nutritional reward for the ants while offering the aphids persistent protection from predators.

Beyond providing protection and nutritional rewards, mutualistic interactions can also indirectly alter competitive dynamics among other herbivorous competitors sharing the same resource. A recent study has demonstrated that *S. invicta* mediates competition between the mealybug *Planococcus lilacinus* and the oriental fruit fly *Bactrocera dorsalis* through chemical communication [93]. In orchard ecosystems, the fruit fly typically holds a competitive advantage over mealybugs when sharing the same food resources. However, when *S. invicta* is present, the situation is reversed, with mealybugs gaining a competitive advantage by attracting *S. invicta* workers to provide protection [93]. *S. invicta* workers can be attracted by honeydew volatiles of the mealybug and, in return for a reward, workers defend the mealybug by depositing semiochemicals such as d-limonene and dodecanoic acid on fruit surfaces, which deter oviposition of the competitor fruit fly [93]. This interaction enhances the persistence of mealybug populations under competitive pressure and highlights the role of *S. invicta* in shaping interspecific dynamics through chemical communication. It also provides potential directions for developing biological strategies against fruit tree pests based on chemical communication.

### 5.2. Interactions with Natural Enemies and Pathogens

Despite their ecological advantage in the invaded habitat, *S. invicta* still faces pressure from natural enemies and pathogenic microorganisms. The social pheromone that underlies the competitive dominance of *S. invicta* can be exploited by its natural enemy as a reliable cue. The phorid fly *Pseudacteon tricuspis*, one of the most well-known specialized natural enemies of fire ants, can be strongly attracted to the alarm pheromone component, particularly 2-ethyl-3,6-dimethylpyrazine, which is effectively recognized by the phorid fly and serves as a reliable cue for locating workers [95]. When workers detect the presence of phorids, they perform a freeze or flee response, which significantly reduces their foraging efficiency, with the foraging rates may be reduced by 50% or more under phorid pressure [95,96].

In addition to parasitoids, chemical communication also plays a critical role in regulating interactions between *S. invicta* and pathogenic microorganisms. Studies have shown that *S. invicta* can efficiently recognize nestmates infected by the pathogenic fungi *Metarhizium anisopliae* through chemical changes in their CHCs, and remove infected corpses from the nest to prevent pathogen spread. Moreover, workers adjust their defensive responses according to the caste and degree of infection of the affected individuals, demonstrating a flexible strategy of social immunity within the colony [97]. On the other hand, to minimize the risk of fungal infection, *S. invicta* can also evolve a strategy to use soil microbial cues to select nesting sites, thereby reducing the risk of infection with pathogenic fungi. Studies have shown that soils rich in actinobacteria contain significantly fewer pathogenic fungi, whose characteristic odors (mainly geosmin and 2-methylisoborneol) strongly attract newly mated *S. invicta* queens for nesting [98]. By recognizing these odor cues, the queens indirectly select environments with lower pathogen pressure, thereby increasing their survival rates and promoting successful colony founding. These findings highlight how the activity of *S. invicta* is shaped by semiochemical-mediated cooperative and antagonistic interactions with other species and provide insights for developing semiochemical-based biological control strategies against *S. invicta*.

## 6. Pheromone Applications in Pest Management

In recent years, pheromone- and semiochemical-based behavioral regulation has increasingly been explored as a critical complementary component of integrated pest management (IPM) for *S. invicta*. In principle, these strategies aim to manipulate collective behaviors of ants by exploiting or interfering with pheromone communication, thereby improving toxic bait delivery, disrupting foraging and recruitment, and strengthening monitoring for early detection and warning.

### 6.1. Pheromone-Enhanced Poisoning Baits

Conventional control of *S. invicta* has mainly relied on insecticides and toxic baits [99]. However, these methods have notable limitations, including environmental concerns, non-target effects, and insufficient long-term suppression efficacy [99,100]. To improve the specificity and efficiency of conventional toxic baits, pheromone-enhanced toxic baits that incorporate trail or alarm pheromones of *S. invicta* into bait formulations have been explored as an ecologically compatible strategy to manipulate foraging behavior and optimize toxicant delivery within colonies [101]. The addition of synthetic pheromone components is intended to enhance the efficiency of worker recruitment and bait discovery. For example, a study evaluated the behavioral effects of multiple candidate pheromone components combined with toxic baits in laboratory and field assays, showing that pheromone application significantly reduced the time required for bait discovery and colony-wide delivery [101]. The trail- and alarm-pheromone-enhanced baits are more attractive to *S. invicta* foraging workers than 13 commercial baits. Specifically, the trail pheromone allofarnesene cis/trans mixture was identified as a promising, high-efficacy attractant, achieving an attraction rate of 68% at low concentrations [5].

In addition to trail and alarm pheromones, introducing food-related semiochemicals into baits is another important approach to enhancing bait attractiveness. A more recent study identified and optimized a food-related blend that significantly increased the orientation and residence time of workers on baits [5]. Taken together, these works indicate significant advantages of pheromone-enhanced baits for more efficient and environmentally compatible control of *S. invicta*. However, several issues still limit their large-scale application, including variation in odor preference among different colonies, the chemical stability of pheromone attractants, controlled release under field conditions, formulation costs, and interference from complex environments. These challenges highlight the importance of further research on screening active attractants, developing slow-release carriers, and optimizing field application strategies before pheromone-enhanced baits can be applied on a large scale in pest management.

### 6.2. Pheromone-Based Monitoring Baits

Accurate monitoring of *S. invicta* is critical for the early detection and effective management. Traditional monitoring methods primarily rely on food-based baits (e.g., sausage or lipid- and protein-rich bait) and ground surveys of nest activity [102]. However, these methods have several limitations, including low efficiency and limited species specificity [103]. In recent years, pheromone-based monitoring approaches have emerged as a promising research direction, as worker activity in *S. invicta* is highly dependent on chemical communication, with alarm and trail pheromones functioning as key signals mediating trail-following and recruitment. Studies have shown that workers exhibit clear dose-dependent responses to both the trail pheromone component, allofarnesene, and the alarm pheromone, 2-ethyl-3,6-dimethylpyrazine. At low dosages, these compounds enhance foraging and recruitment activity, thereby accelerating bait discovery by workers [101]. Accordingly, integrating pheromones with food-based baits is expected to improve both the sensitivity and specificity of monitoring. Overall, although the stability and effectiveness of pheromone-based monitoring at large scales still require further validation, the pheromone-based strategy provides a reliable basis for precise monitoring and early warning of *S. invicta*.

### 6.3. Pheromone Disruption of Foraging and Recruitment

Alternatively, pheromone disruption techniques typically involve applying an overdose of synthetic pheromones into the environment or at inappropriate locations, thereby disrupting chemical communication, orientation, and recruitment of ants, and ultimately suppressing foraging and resource acquisition. Trail pheromone of *S. invicta* has been used in early studies to evaluate the feasibility of such techniques. Laboratory work assessed the effects of high concentrations of Z,E-α-farnesene, one of the key components of *S. invicta* trail pheromone, on the orientation and foraging efficiency of workers [104]. The results showed that the time taken by workers to locate the food source and transport bait increased significantly, and the success rate of individuals returning to the nest with food decreased markedly. In another work, this research group further confirmed the disruptive effect of Z,E-α-farnesene by aerosol delivery [73].

These results indicate that pheromone disruption of foraging and recruitment in *S. invicta* is theoretically feasible. However, current evidence is mainly derived from laboratory or semi-field experiments, while the disruption effect under complex field conditions has not yet been systematically evaluated. In addition, the application of this strategy is limited by several practical issues, including the cost of synthetic pheromone and formulation stability. In particular, the production of high-purity Z,E-α-farnesene remains technically challenging, and the cost is relatively high [73]. These items together limit the large-scale application of pheromone disruption strategies.

## 7. Research Gaps and Future Prospects

### 7.1. Pheromone Identity and Signal Processing Mechanisms

Although pheromones play central roles in regulating social organization and collective behaviors in *S. invicta,* their specific chemical identities, signal statuses, and the underlying processing mechanism remain poorly understood. As discussed in Section 4.2, most evidence for queens regulating worker behavior and reproductive physiology is primarily derived from behavioral observations or assays using glandular or cuticular extracts; however, the active components and their roles as social signals have not been systematically defined. For instance, a blend of three queen-associated compounds has been reported to induce worker aggregation or retinue responses under experimental conditions [24,29]. However, these effects alone do not demonstrate that these compounds function as dedicated signals of queen identity or reproductive status under natural conditions. To address these issues, future studies should primarily focus on characterizing the active components, the natural source, and the behaviorally active dose of the candidate pheromone, and on demonstrating the necessity and sufficiency of the chemical cues in mediating queen recognition.

In terms of pheromone sensory mechanisms in *S. invicta*, one of the most significant gaps concerns the functional characterization of ORs. As discussed in Section 3.1, genomic analyses have revealed a substantial expansion of OR repertoire, and the expansion of 9-exon OR lineage has been proposed to relate to the detection of social pheromones [57,58]. However, experimental evidence directly linking specific ORs to ecologically relevant pheromone components is largely lacking. This gap limits our understanding of central questions about how specific ORs mediate pheromone communication and whether the expansion of OR repertoire contributes to social behavior regulation and further promotes ecological adaptation of *S. invicta*. Therefore, future studies should focus on systematic functional characterization of OR repertoire to establish direct links between OR functional diversification and species-specific behaviors mediated by pheromones.

Beyond peripheral receptor-ligand interactions, the neural coding strategies underlying pheromone information processing in *S. invicta* remain largely unresolved. To date, a comprehensive structural and functional map of the primary olfactory processing center (e.g., the AL) has not been established. Consequently, it remains unclear whether key pheromones are processed through dedicated labeled-line pathways, combinatorial coding, or a combination of both. Resolving these questions is essential to illustrating how specific pheromones regulate social behaviors and the context-dependent flexibility of their regulation in *S. invicta*. Together, addressing these issues will also provide a critical scientific foundation for the development of pheromone-based behavioral regulation strategies.

### 7.2. Chemical Ecology of Intra- and Interspecific Interaction

As discussed in previous sections, pheromones and other semiochemicals regulate both intraspecific behaviors and interspecific interactions in *S. invicta*. Importantly, the chemical communication system underlying these interactions is dynamic rather than fixed, exhibiting marked variation and plasticity across social and ecological contexts. In particular, it remains unclear whether pheromone composition, perception mechanisms, and colony-level signal representation differ between monogyne and polygyne colonies. Moreover, it is essential for understanding whether such differences contribute to divergence in social organization and collective behavior, thereby facilitating an understanding of the evolutionary dynamics of social systems in *S. invicta*. Recent evidence that heterozygous SB/Sb workers can induce a transition in social forms (Section 4.4) provides an important advance in this area, highlighting the potential role of chemical signals not only in reflecting social structure but also in driving social reorganization [30].

In interspecific interactions, as previously discussed in Section 5, semiochemicals play a central role in mediating interactions between *S. invicta* with organisms across multiple trophic levels, including mutualists, competitors, and natural enemies. However, such interactions are often highly context-dependent, as specific chemical cues and their functional outcomes can vary across ecological conditions, developmental stages, and interacting species. Therefore, future studies should more systematically identify the key chemical signals that mediate multiple trophic level interactions and evaluate their functional stability across diverse ecological contexts. Together, these studies provide important insights into the ecological adaptation of *S. invicta* and offer a foundation for developing biological regulation strategies based on chemical communication.

### 7.3. Pheromone-Based Behavioral Regulation for Management of S. invicta

Accumulating studies have demonstrated the potential of pheromone-based behavioral regulation strategies, which exhibit high sensitivity and strong species specificity. However, their application in large-scale field settings remains limited. For instance, foraging and recruitment disruption based on trail pheromones have been validated primarily in laboratory or semi-field settings. As discussed in Section 6.3, one major limitation is the high cost of large quantities of purified compounds. In addition, trail pheromone signals function as short-range and short-term signals under natural conditions, whereas effective disruption strategies rely on sustained signal output [105]. Beyond these technical limitations, the mechanistic basis underlying trail pheromone regulation remains poorly understood. In particular, it is unclear whether and how workers rely on trail pheromones differs across social forms and geographic populations, and whether such behavioral disruption is subsequently amplified to influence colony-level population dynamics.

Future directions will therefore require advances in several key areas. From a technical perspective, a primary focus should be on optimizing pheromone synthesis and blend composition to enhance biological activity, as well as developing controlled-release systems suitable for variable field conditions. In parallel, mechanistic and ecological studies are equally critical for linking pheromone-mediated behavioral effects to colony-level outcomes. At the application level, large-scale field trials with long-term assessments are required to evaluate the impacts on colony population dynamics and potential effects on non-target organisms across different habitats. Within the IPM framework, it is further necessary to assess synergies with other management tools, particularly through integration with emerging monitoring technologies, such as rapid identification via machine learning and real-time monitoring. Overall, pheromone-based behavioral regulation provides a promising strategy for the management of *S. invicta*.

## Data Availability

No new data were created or analyzed in this study. Data sharing is not applicable to this article.
